# Agreement between Clinical Frailty Scale-scores based on information from patient interviews and Clinical Frailty Scale-scores based on information from medical records - a cross sectional study

**DOI:** 10.1186/s12877-024-05160-5

**Published:** 2024-07-02

**Authors:** Kim Jackwert, Michael Holmér, Matilda Hallongren, Todel Asmar, Per Wretenberg, Åsa G Andersson

**Affiliations:** 1 School of Medical Sciences, Faculty of Medicine and Health, Örebro University, Örebro, SE70182 Sweden; 2 Department of Geriatrics, School of Medical Sciences, Faculty of Medicine and Health, Örebro University, Örebro, Sweden; 3 Department of Orthopaedics, School of Medical Sciences, Faculty of Medicine and Health, Örebro University, Örebro, Sweden

**Keywords:** Frailty, Geriatric, Dementia, Clinical frailty scale, Agreement, Medical records

## Abstract

**Introduction:**

Frailty is an age-related condition with increased risk for adverse health outcomes. Assessing frailty according to the Clinical Frailty Scale (CFS) based on data from medical records is useful for previously unassessed patients, but the validity of such scores in exclusively geriatric populations and in patients with dementia is relatively unknown.

**Methods:**

Patients admitted for the first time to one of two geriatric wards at Örebro University hospital between January 1st – December 31st, 2021, were included in this study if they had been appointed a CFS-score by anamnestic interview (CFS_I_) at admission. CFS scores based on medical records (CFS_R_) were appointed by a single medical student, who was blinded to the CFS_I_ score. Score-agreement was evaluated with quadratic weighted Cohen’s kappa (*κ*).

**Results:**

In total, 145 patients between the age of 55–101 were included in the study. The CFS_R_ and CFS_I_ scores agreed perfectly in 102 cases (0.7, 95% CI 0.65–0.77). There was no significant difference regarding age, sex, comorbidity, or number of patients diagnosed with dementia between the patients with complete agreement and the patients whose scores did not agree. Agreement between the scores was substantial, *κ* = 0.66, 95% CI 0.53–0.80.

**Conclusions:**

CFS scores based on information from medical records can be generated with substantial agreement to CFS scores based on in-person anamnestic interviews. A dementia diagnosis does not influence the agreement between the scores. Therefore, these scores are a useful tool for assessing frailty in geriatric patients who previously lack a frailty assessment, both in clinical practice and future research. The results support previous findings, but larger studies are warranted.

## Introduction

Old age poses an increased risk for frailty, thereby, as the general population of the world is getting older, the prevalence of frailty increases [1–3]. Frailty poses an increased risk to adverse health outcomes such as falls, hospitalization, admission to nursing homes and mortality [1]. It may also affect the response to treatments and recovery of mobility in older patients [4].

There is currently no consensus definition for frailty. The condition is commonly described as a multifactorial state or syndrome of higher risk for health issues due to a reduction in biological reserves and the ability of the body to adapt and resist external stressors. As the ability to adapt is compromised, this creates vulnerability [1, 2, 5]. Frailty and the lack of adaptive capacity is closely related to ageing [1, 3], but may arise from other conditions such as systemic illnesses or severe injuries [2].

Since there is no general definition for frailty, there also is no standard tool for measuring or grading frailty [6]. Grading frailty is important for risk stratification as well as for diagnosing and health care planning, such as to identify patients who would benefit from a comprehensive geriatric assessment (CGA) [7, 8]. The Clinical Frailty Scale (CFS) is a widely used, easily applicable tool for measuring frailty and has shown good predictive ability and high inter-rater reliability [9–11]. The CFS, developed in 2005 by Rockwood et al., considers matters of both physical and mental frailty [5]. It was later expanded to include guidelines on how to score patients with dementia [10]. Dementia significantly influences several aspects evaluated by the CFS, which complicates its use as a frailty assessment tool.

The CFS can be a component of a comprehensive geriatric assessment and is commonly used during consultations with patients or their caregivers. To achieve individualized care, the department of Geriatrics at Örebro University Hospital decided in the year of 2021 to assess frailty by means of the CFS on all patients on admittance to the clinic. Because of several reasons, staff shortage among others, these assessments could not be carried out as planned in clinical everyday practice. To produce data on possible frailty for patients who were not assessed on admittance, frailty assessments according to the CFS needed to be conducted in retrospect based on information from medical records. Several studies have previously demonstrated the validity of CFS scores based on data from medical records [11–14]. However, there is still insufficient data to confirm the validity of these CFS scores, especially in geriatric populations and in patients with dementia.

The aim of this study is to examine agreement between CFS scores acquired from studying medical records (CFS_R_) and CFS scores determined by hospital staff through anamnestic interviews at admission (CFS_I_), along with the possible impact on this agreement by a dementia diagnosis.

## Methods

### Study design and population

This is a cross sectional study evaluating patients admitted to two separate wards at the department of Geriatrics at Örebro University Hospital. Patients were included in the study if they were admitted for the first time between January 1st – December 31st, 2021, and had been appointed a CFS_I_ score.

### Collection of data

Frailty was assessed according to a verified Swedish translation of the CFS version 2.0 [15]. The CFS 2.0 is an ordinal scale ranging from 1 (very fit) to 9 (terminally ill). Scoring is based on the patient’s baseline state, which is usually defined as their state two weeks prior to assessment [10]. Patients with a score of CFS > 5 were considered frail. The CFS_I_ score for each patient had been appointed at admission to the Geriatric wards according to a routine frailty assessment. This assessment consists of an interview with the patient and, sometimes, their close relatives, friends, or caregivers, and is conducted by a nurse, occupational therapist, or physiotherapist. The CFS_R_ scores were generated through a systematic review of each patient’s medical records. This was conducted by a single medical student, who was blinded to the CFS_I_ scores. Record entries from nurses, occupational therapists, physiotherapists, and physicians, dating at least 14 days prior admission, were reviewed. The CFS_R_ scores were then based on information about the patient’s activities of daily life (ADL), cognition, living situation, medical history, and physical abilities. In cases of uncertainty about a score, the higher score was appointed. At two occasions, the medical student accidentally saw the CFS_I_ score in the records. The patients in question were therefore not included from the study. The CFS_R_ score was subsequently compared to the CFS_I_ score.

#### Measurement

A customized standardized form designed for the purpose of this study was used to obtain relevant patient characteristics. The form included the patients’ gender, birthyear, and primary and secondary diagnoses based on the ICD-10. The latter were required to calculate the Charlson Comorbidity Index (CCI), which was used to weigh the patients’ comorbidities. The CCI was developed by Charlson et al. in 1987 and later adapted for register-based research in Sweden by Ludvigsson et al. The index is used to weigh comorbidities to estimate one-year mortality and consists of a numeric scale based on the presence of specific diagnoses as well as the patient’s age [16]. A higher score on the CCI indicates a higher one-year mortality [17]. A patient’s primary diagnosis was defined as the diagnosis set as main diagnosis on admission to the geriatric ward. Any other diagnoses were defined as secondary diagnoses. To portrait patient characteristics in Table 1, the primary diagnoses were sorted into six categories according to prevalence: orthopaedic, dementia, respiratory, cardio-vascular, surgical, and other. Any diagnosis not among the first five most prevalent categories was included in “others”.

**Table 1:** Patient characteristics, *n* = 145

Age, mean (SD) years		82 (9)
Range		55–101
Male, *n* (%)		79 (54)
Living situation, *n* (%)		
	Ordinary housing	130 (90)
	Nursing home	15 (10)
Main Diagnosis (ICD-10), *n* (%)		
	Dementia	21 (14)
	Surgical	20 (14)
	Cardio-Vascular	18 (12)
	Orthopaedic	16 (11)
	Respiratory	15 (10)
	Other*	55 (38)
Charlson Comorbidity Index, CCI, median (IQR)		5 (4–6)
Range		2–10
Frail, Clinical Frailty Scale > 5, *n* (%)		132 (91)

SD = Standard deviation, IQR = Interquartile Range

*Includes all diagnoses not among the five most prevalent categories of diagnoses

### Statistical analysis

To analyze differences in age and comorbidities by means of the CCI, the Unpaired t-test and Mann-Whitney U-test were utilized respectively. The Chi-squared test was used to analyze differences in gender and dementia diagnoses. Agreement between the CFS_I_ and CFS_R_ scores was measured with paired-sample sign test and quadratic weighted Cohen’s kappa (*κ*). The *κ*-value was interpreted according to Viera et al. [18]. The level of statistical significance was set to *p* < 0.05. Statistical analyses were performed using IBM SPSS Statistics for Windows, version 29.0, Armonk, Ny: IBM Corp.

The method described by Newcombe & Altman [19] was used to calculate the 95% confidence interval (CI) for the proportion of CFS scores with perfect agreement. Results were deemed significant if the 95% CI did not include zero. The analysis was conducted using the software Confidence Interval Analysis version 2.2.0, University of Southampton.

### Ethical considerations

All collected personal data was encoded and stored in a locked file cabinet at the department of Geriatrics. The study is approved by the Swedish Ethical Review Authority (Dnr 2022-01360-01 and dnr 2023-00994-02) and no written consent was required from the patients.

## Results

**Fig. 1:**
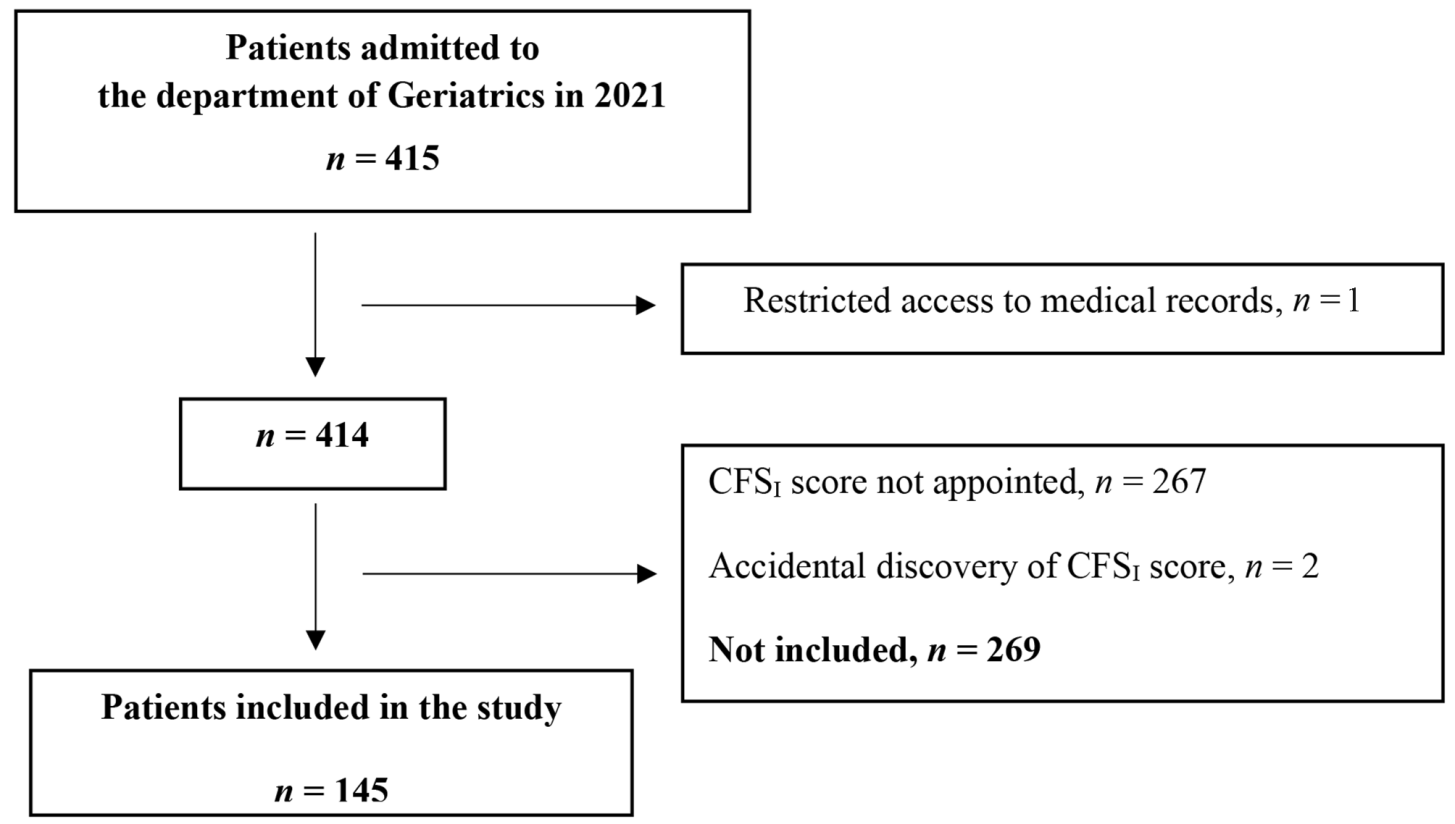
Flow chart of the study inclusion process. CFS_I_ score = Clinical Frailty Scale score appointed by hospital staff on admission based on information from anamnestic interviews

In the year of 2021, 415 patients were admitted to the department of Geriatrics at Örebro University Hospital. Of these, 269 patients were not included because they either had not been appointed a CFS_I_ score on admission, because of restricted access to their medical records, or due to accidental discovery of the CFS_I_ score while reading their medical records (Fig. 1). There was no significant difference in gender, age or comorbidity between the patients included and the patients not included in the study (*p* = 0.23, *p* = 0.21 and *p* = 0.14 respectively). Patient characteristics are presented in Table 1. Apart from the patients having a dementia diagnosis as their main diagnosis, as seen in Table 1, several other patients were diagnosed with dementia as a secondary diagnosis. In total there were 30 patients (21%) with a dementia diagnosis enrolled in this study.

### Primary outcome- agreement between the CFS_I_- and CFS_R_-scores

All generated CFS_I_ and CFS_R_ scores are presented in Table 2. The two scores agreed perfectly in 102 of 145 cases (0.7, 95% CI 0.65–0.77). In 12 cases, the CFS_R_ score was higher than the CFS_I_ score, and in 31 cases it was lower than the CFS_I_ score. CFS 7 was the most appointed score. No patient was appointed a score of 1 (very fit) or 2 (fit). CFS 3 and 9 were only appointed to one patient respectively. A comparatively low prevalence was also seen for CFS 4 and 8. The median CFS_R_ score was 6 (IQR 5–7) and the median CFS_I_ was 7 (IQR 6–7), *p* < 0.005. Agreement between the CFS_R_ and CFS_I_ scores was substantial (*κ* = 0.66, 95% CI 0.53–0.80).

**Table 2:** Agreement between interview-based scores (CFS_I_) and medical record-based scores (CFS_R_). Total scores, *n* = 145

	CFS_I_						
	CFS-score∗	4 (very mild frailty)	5 (mild frailty)	6 (moderate frailty)	7 (severe frailty)	8 (very severe frailty)	9 (terminally ill)
CFS_R_	3 (managing well)	0	0	0	1	0	0
	4 (very mild frailty)	**6**	3	1	2	0	0
	5 (mild frailty)	1	**21**	9	3	0	0
	6 (moderate frailty)	1	2	**20**	9	0	1
	7 (severe frailty)	0	2	4	**54**	2	0
	8 (very severe frailty)	0	0	1	1	**1**	0

Bold numbers represent scores with perfect agreement between CFS_R_ and CFS_I_

CFS = Clinical Frailty Scale

∗ No patients were appointed a CFS_I_ score of 1, 2, 3, or a CFS_R_ score of 1, 2, 9

### Secondary outcome- impact of dementia on agreement

There was no significant difference in age, comorbidity, gender or the prevalence of dementia between the patients for whom the CFS_I_ and CFS_R_ scores agreed and the patients for whom the scores did not agree (Table 3).

**Table 3:** Analysis of possible effect on score agreement by patient characteristics. Patients, *n* = 145

	Agreed *n* = 102	Did not agree *n* = 43	*p*-value
Age, mean (SD), years	82 (9)	83 (9)	0.49
Male, *n* (%)	57 (56)	22 (51)	0.60
Charlson Comorbidity Index, CCI, median (IQR)	5 (4–6)	5 (4–6)	0.19
Dementia, *n* (%)	24 (24)	6 (14)	0.19

SD = Standard deviation, IQR = Interquartile Range

## Discussion

This study examined 145 patients who were admitted to the department of Geriatrics at Örebro University hospital during 2021. The aim was to examine agreement between CFS scores acquired from studying medical records (CFS_R_) and CFS scores determined by hospital staff through anamnestic interviews at admission (CFS_I_), along with the possible impact on this agreement by a dementia diagnosis. Perfect agreement between the scores was seen in 102 of 145 cases (70%). There was no significant difference regarding age, sex, comorbidity, or number of patients diagnosed with dementia between the 102 patients with complete agreement and the 43 patients whose scores did not agree.

This study shows a substantial agreement between CFS_I_ and CFS_R_ scores, which is in accordance with previous studies [11–14]. For two of these studies, a direct comparison cannot be made due to differences in the methods used for presenting agreement between the scores [11, 14]. Two other studies presented their results by means of Cohen’s kappa, as was done in this study [12, 13]. As one of these studies did not specifically use weighted Cohen’s kappa, a direct comparison may again not be possible [12]. The second study, a German study from 2020 including 110 patients, presented a weighted Cohen’s kappa value of 0.89, which represents an excellent agreement [13]. Compared to the value presented in this study, which was 0.66 and represents substantial agreement, the German study showed a higher rate of agreement. It is possible that this difference can be attributed to the different strategies used for appointing raters. Compared to this study, the German study had three staff members in total rating all scores, albeit being blinded to the individual patient’s other CFS score [13]. It is possible that fewer raters lead to higher agreement since it minimizes the variabilities in experience and methods applied by different people.

The score with best agreement between the CFS_I_ and CFS_R_ scores was CFS 7. This may be attributed to the distinct criteria set for CFS 7 by Rockwood et al. [10], which may have been easier to recognize in a patient. Determining agreement for the lowest and highest scores of the CFS was difficult as the prevalence of these scores was low, as seen in Table 2. Only a few patients were appointed a score of CFS 3, 8 or 9, and no patient was appointed a score of CFS 1 or 2. The distribution of scores shown in Table 2 is a credible reflection of the generally frail, but not terminally ill, population which is at higher risk to be admitted to a geriatric ward in the next 14 days. Since the agreement for the lowest and highest scores could not be correctly determined, it is possible that it may be either lower or higher compared to the agreement for the scores closer to the middle of the scale. A lower agreement would be a disadvantage for affected patients, as treatment based on their CFS_R_ score would likely be insufficient or inappropriate. Comparing agreement for these scores specifically would be relevant to study in the future, particularly as they have had a low prevalence in previous studies as well [11, 12].

This study showed a statistically significant tendency to produce lower CFS scores when using information from medical records compared to when using information from anamnestic interviews, and therefore deeming patients as being less frail when using medical records (*p* < 0.005). The same tendency was observed in a German study from 2020, however, it was deemed clinically insignificant [13]. Perhaps a reason for this tendency may be that a person appointing a CFS score by means of studying medical records is less likely to be biased by the patient’s current condition as the patient is not present at the time of assessment. This would suggest that, in those cases where the CFS_R_ and the the CFS_I_ scores differ, the CFS_R_ score may more accurately represent the patient’s frailty status, as the patient’s condition at the time of assessment for the CFS_I_ is likely worse than their habitual condition due to the current hospitalization. Another reason for appointing lower CFS_R_ scores may have been outdated information in medical records, which would instead result in a probable underestimation of frailty by CFS_R_ scoring. In contrast, a study from 2022 showed a tendency for CFS_R_ scores to be higher than CFS_I_ scores, but the difference was not statistically significant [14]. Until sufficient research can rule out a possible tendency to over- or underestimate frailty through CFS_R_ scores, they should only be utilized if the patient was for some reason not assigned a CFS_I_ score but a score is needed for research or as guide to deciding further treatment. This minimizes the risk of excluding a potential patient from treatment that is reserved for patients with a certain CFS score, such as CGA.

Applying the CFS on patients with a dementia diagnosis may prove to be especially difficult as information on the patient’s physical and mental health state may be more difficult to obtain. This study found no significant difference in prevalence of a dementia diagnosis between the patients for whom the CFS_I_ and CFS_R_ scores agreed and the patients for whom the scores did not agree. This suggests that a dementia diagnosis does not influence the accuracy of CFS_R_ scores, indicating that they may be used for patients with dementia as well.

Generating CFS scores solely based on information in medical records is dependent on the quality of the records. In those cases where patients have not been recently hospitalized, information is scarce and appointing an appropriate score is difficult. However, if a patient has been treated recently, the information provided is detailed and well suited for CFS-scoring. As the quality of medical records may vary throughout medical institutions, this may limit the generalisability of this study. This limitation does not apply when generating CFS scores based on in-person interviews as information about the patient’s health status 14 days prior to admission is obtained first hand, with the possibility to complete any missing information by further asking the patient’s close family, friends, or caregivers. The quality of information with this approach is instead dependent on the person conducting the interview, wherefore adequate training in CFS-scoring is essential. As in-person interviews pose more advantages, CFS_R_ scores should not replace CFS_I_ scores, only complement them.

A limitation of this study is its small patient sample. This was mainly due to it being a single centre study and due to the major drop out resulting predominantly from the lack of CFS_I_ scores. The drop out itself may have been a weakness, although drop out analysis did not show any significant differences in age, gender or CCI between the inclusion group and the patients not included. Additionally, as CFS_I_ scores were appointed by different hospital staff, equal assessment of all patients cannot be guaranteed. Based on recent studies, which showed high inter-rater agreement between CFS scores [11, 13, 14], this was deemed to not significantly have affected the study outcome. Another possible limitation regarding the CFS_I_ scores is recall bias and not taking in count that the scores reflect the patients state of health 14 days prior to the assessment day. A possible bias for this study design would be basing the CFS_R_-scores on record entries containing information from the anamnestic interview conducted on admission for assessing the CFS_I_-scores. This bias was avoided by basing the CFS_R_-scores solely on information from record entries dated earlier than the patients’ admission to the department of Geriatrics.

A strength seen in this study was that all CFS_R_ scores were appointed by the same person. Together with the use of the standardized forms employed for each patient, this ensured equal assessment of all patients in regard to CFS_R_ scoring. In addition, the medical student appointing these CFS scores had no contact with the hospital staff appointing the CFS_I_ scores or the patients involved. This ensured that the student was completely blinded to the CFS_I_ score and to information about the patients outside of the medical records. However, the fact that only one person appointed all CFS_R_ scores may also be a weakness.

The drop out seen in this study due to missing CFS_I_ scores illustrates the need for CFS_R_ scoring in clinical settings. As frailty is related to adverse health outcomes and may affect treatment response or recovery of mobility in elderly patients [1, 4], evaluating the possibility of frailty in geriatric patients is of great importance. Implicating CFS_R_ scores for patients who, for several reasons, were not appointed a CFS_I_ score on admission is therefore an important measure.

## Conclusions

CFS scores based on information from medical records can be generated with substantial agreement to CFS scores based on in-person anamnestic interviews. A dementia diagnosis does not influence the agreement between the scores. Therefore, these scores are a useful tool for assessing frailty in geriatric patients who previously lack a frailty assessment, both in clinical practice and future research. The results support previous findings, but larger studies are warranted.

## Data Availability

The datasets generated and analysed in this study are not publicly available due to ethical restrictions and laws (GDPR) of disclosing personal data. Inquiries for data access should be sent to the corresponding author, asa.andersson@oru.se, who will then contact the ethics board at Örebro University for permission to openly share the data.

## Abbreviations

ADL: Activities of daily life
CCI: Charlson Comorbidity Index
CFS: Clinical Frailty Scale
CFS_R_: Clinical Frailty Scale score based on medical records
CFS_I_: Clinical Frailty Scale score based on anamnestic interviews
CGA: Comprehensive Geriatric Assessment
CI: Confidence interval
FI: Frailty Index
ICUs: Intensive Care Units
